# Exploring the Mechanism of Microstructural Changes in Ultra-High-Performance Concrete Under Microwave Influence: Experiments and Molecular Dynamics Simulation

**DOI:** 10.3390/ma18091892

**Published:** 2025-04-22

**Authors:** Jingyuan Chen, Kunyang Yu, Shuangxin Li, Dengao Liu

**Affiliations:** 1 School of Civil Engineering and Transportation, Northeast Forestry University, Harbin 150060, China; 2 School of Water Conservancy and Civil Engineering, Northeast Agricultural University, Harbin 150030, China; 3 School of Civil Engineering and Architecture, Zhejiang University of Water Resources and Electric Power, Hangzhou 310018, China

**Keywords:** microwave curing, microstructure, UHPC, C-S-H, molecular dynamics

## Abstract

To elucidate the mechanisms of microstructural changes in ultra-high-performance concrete (UHPC) under microwave exposure, this study characterizes the microstructure at multiple scales using a combination of microscopic experiments and molecular dynamics simulations. The hydration products, pore structure, morphology, and interface transition zone (ITZ) of UHPC specimens were analyzed using mercury intrusion porosimetry (MIP), X-ray diffraction (XRD), and scanning electron microscopy (SEM). Molecular dynamics simulations were employed to investigate the uniaxial tensile behavior, free volume, and radial distribution of calcium silicate hydrate (C-S-H) gel, the primary hydration product. The results indicate that microwave curing significantly reduces the pore volume of specimens, with a daily average reduction of 0.15% in the early stages. This accelerated reduction in porosity effectively diminishes the number of high-risk pores. The hydration products formed under microwave curing exhibit higher density and enhanced internal pore optimization. Simulation findings suggest that the non-thermal effects of microwaves play a more significant role in the structural evolution. The molecular orientation of C-S-H changes after oscillation, leading to more ordered molecular arrangements. Mechanical oscillation also expels free volume from the crystal cells, promoting a more compact overall structure and increasing the tensile strength by up to 1 GPa.

## 1. Introduction

A product of today’s rapidly developing engineering field, UHPC, a cutting-edge cement-based material, has demonstrated tremendous application potential in bridges, highways, airports, and marine engineering due to its excellent strength, impact resistance, and remarkable durability [1,2], earning high praise from engineers and researchers. However, the early compressive strength of UHPC matrices cured under normal temperature conditions often struggles to exceed 120 MPa [3]. To address this challenge, scholars are exploring accelerated curing methods to expedite its early reaction and enhance its mechanical properties. Therefore, delving deeply into the mechanism of microscopic structural changes in rapidly cured concrete holds significant theoretical importance for assessing the accuracy of performance predictions [4], improving the effectiveness of mechanical properties [5], and optimizing material design [6].

Microwaves, as a clean heating technology, offer advantages such as rapid heating, high energy efficiency, uniform heating throughout the material, and easy control [7]. They have been studied in relation to the rapid curing of concrete materials. With the increasing industrialization of the construction industry, the application of precast concrete in construction has become more frequent. Compared to traditional heating curing methods, microwave curing consumes only 10% of the energy [8]. Numerous scholars have initiated detailed and comprehensive research on this construction technology. Studies have shown that microwave volumetric heating effectively resolves thermal stress issues caused by temperature gradients, promoting the formation of a denser and more uniform ITZ [9]. Additionally, the evaporation of free water reduces capillary pores, leading to a corresponding decrease in porosity, which contributes to a denser and stronger concrete structure [10]. Li et al. [11] found that using low-power and low-temperature microwave curing methods can prevent thermal damage in coal gangue concrete. Liu et al. [12] pointed out that microwave pre-curing technology enhances the packing density of C-S-H gel, optimizing the creep properties of concrete. Shen et al. [13] and Xia et al. [14], respectively, discovered that in ultra-high-performance geopolymer concrete and lime-calcined clay cement mortar, microwave curing technology promoted the tight accumulation of gel, refined the pore network, significantly reduced porosity, and enhanced the overall density of the material. Furthermore, Li et al. [15,16] conducted a comparative analysis of young and aged samples that underwent delayed microwave curing, delving into the short-term and long-term effects of microwave curing on the micro- and nano-structures of cement and slag pastes.

Researchers have achieved meaningful results when studying the enhancement of concrete properties through microwave curing, providing a basis for understanding and improving concrete microstructure and mechanical properties. However, few studies have investigated the impact of microwaves on concrete’s structural characteristics at the molecular level.

Over the past few decades, breakthroughs in nanotechnology have made it possible to study complex phenomena in nanoscale material systems. Material deformation always originates at the atomic scale, which is often overlooked when using traditional techniques, including existing experimental tests and continuum theory. Such barriers impeded the development of building materials until the concept of nanotechnology (through simulation) emerged as an effective method, truly breaking the deadlock in the field of building material research. More importantly, the application of nanotechnology in building materials can help us discover various fundamental failure mechanisms within material systems [17], providing much-needed inspiration for mechanistic analysis that extends beyond the scope of building materials. C-S-H, as a key component of cement hydration products, has a decisive influence on the performance of mortar and concrete [18]. In recent years, research on C-S-H has become a hot topic [19]. With the rapid advancement of nanoscale characterization techniques and material computational simulation techniques, many domestic and international scholars have used molecular dynamics simulations to conduct in-depth studies on the mechanical properties [20,21], permeability [22,23], and other aspects of C-S-H gel. This has, to some extent, elucidated the micro-mechanisms and dynamic processes underlying its macroscopic properties. However, there are few studies on the effect of simulated microwaves on the microstructure of cement concrete materials.

This study integrates macroscopic experiments with molecular dynamics simulations to comprehensively and deeply explore the impact of microwave radiation on the microstructure of UHPC specimens from micro- to nanoscales, offering a fresh perspective and insights into the mechanism analysis of microwave curing for UHPC. At the macroscopic experimental level, UHPC samples were prepared and subjected to two curing methods: standard curing and a combined microwave–standard curing approach. Among them, the microwave curing conditions were selected from the most effective one identified in prior research [24], ensuring that the study was based on reliable and optimized parameters. Subsequently, various characterization techniques, such as mercury intrusion porosimetry (MIP) testing, X-ray diffraction (XRD) testing, and scanning electron microscopy (SEM) testing, were employed to conduct a holistic analysis of the samples to obtain detailed information regarding their microstructures. In the realm of molecular dynamics simulations, a microwave field environment was developed, and the established C-S-H model was subjected to dynamic optimization within microwave field environments at varying temperatures. This approach enabled the precise simulation of the dynamic transformation process of the C-S-H structure under microwave influence. The evolution patterns of parameters such as the model’s pore structure, radial distribution function, and tensile strength were compared and analyzed. This study aims to furnish a theoretical basis for optimizing the performance of UHPC and developing microwave curing technologies by gaining an in-depth understanding of the mechanisms through which microwave radiation affects the microstructure and properties of UHPC.

## 2. Materials and Methods

### 2.1. Materials and Preparation

**Figure 1:**
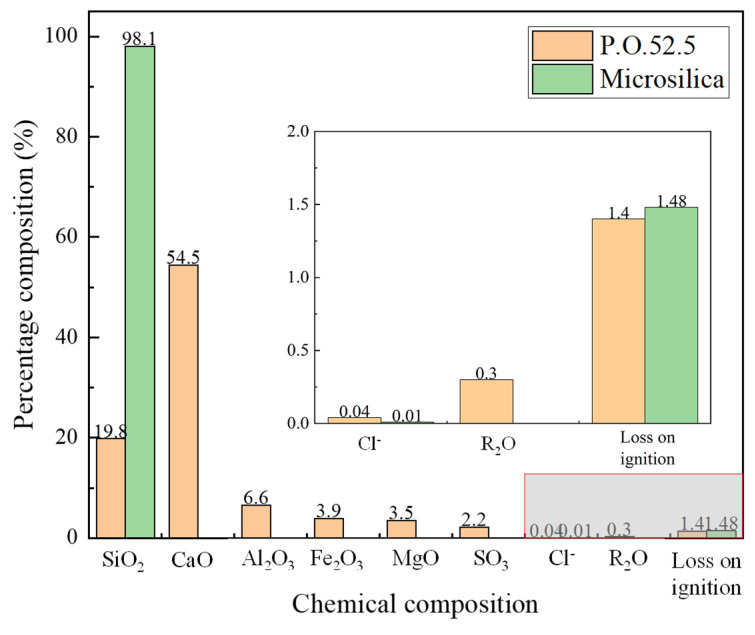
Chemical compositions of P.O.52.5 Portland cement and silica fume [24].

The binder materials used were Portland cement (P.O. 52.5) and silica fume, and their chemical compositions are shown in Figure 1. The specific surface areas of the cement and silica fume were 381 m²/kg and 21,000 m²/kg, respectively. Quartz sand was used as the fine aggregate, and its physical properties are listed in Table 1. The polycarboxylate ether (PCE) superplasticizer used was Sika^®^ ViscoCrete^®^–540P. The water-to-cement ratio was 0.23, and the water-to-binder ratio was 0.18. The mix proportions are detailed in Table 2.

The dry ingredients (cement, quartz sand, and silica fume) were combined according to the predetermined proportions in a JJ-5-type cement mortar mixer and subjected to a 2 min dry mixing process to ensure uniformity. A high-efficiency superplasticizer was thoroughly dissolved in water and added to the mixed dry materials after 2 min. The mixture was then initially blended at a low speed (140 r/min) for 1 min, followed by high-speed blending (285 r/min) for another minute. The paste was carefully poured into 40 × 40 × 40 mm molds and subsequently subjected to either standard curing or microwave curing.

### 2.2. Curing Methods and Regimes

**Figure 2:**
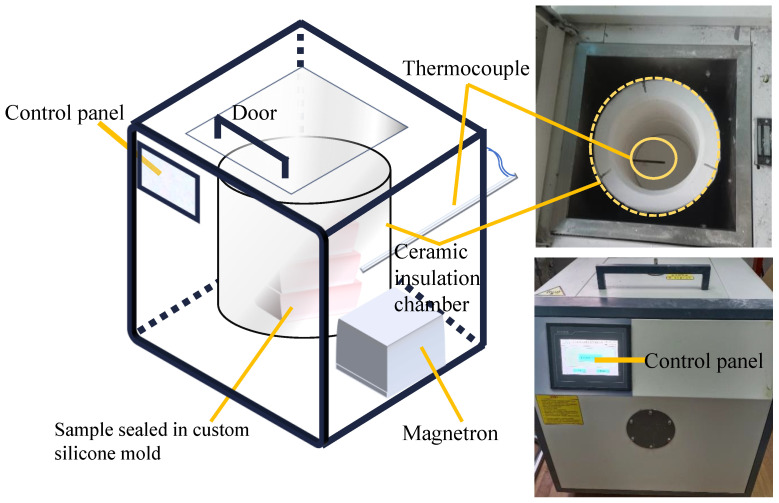
Customized microwave heating apparatus.

To investigate the effects of microwave treatment on UHPC, a standard curing control group was prepared under identical preparation conditions. According to the national standard of the People’s Republic of China, GB/T50081, “Test Methods for Mechanical Properties of Concrete” [25], the standard control group was cured using a standard curing chamber maintained at 20 ± 2°C with a humidity exceeding 95%. A customized microwave heating device (as shown in Figure 2) was utilized to perform rapid curing, where the samples were heated from room temperature to a preset temperature within 10 min, followed by constant-temperature microwave curing. After the microwave pre-curing, the samples were demolded and placed in the standard curing chamber for durations of 1 day, 7 days, and 28 days. The microwave curing regime was selected from previous research results [24]. By testing the daily change rate of the dielectric constant under different curing regimes, it was found that microwave heating at 30°C for 15 min was the most effective in accelerating the hydration rate. This indicates that this regime can more intuitively and clearly present the differential comparison results between microwave curing and standard curing. Therefore, in this study, three groups of specimens were prepared under these microwave curing conditions. After dielectric property tests, one group with data similar to that of a previously studied group was selected as the test sample.

### 2.3. Microstructural Characterization

#### 2.3.1. XRD

After undergoing hydration, the samples were manually ground and sieved through a 200-mesh sieve. XRD analysis was performed using an XRD-6100 X-ray diffractometer from Shimadzu (Kyoto, Japan) to analyze powder from each sample. The scanning range was set from 5° to 80°, with a step size of 0.02° and a step time of 0.15 s.

#### 2.3.2. SEM

A cold field-emission scanning electron microscope, model JME-7500F from JEOL (Tokyo, Japan), was used to observe the cross-sections of the samples under high-vacuum conditions. After the samples were dried, they were attached to conductive adhesive tape on circular mounting stages and subjected to a secondary sputtering of gold to enhance electrical conductivity. The samples were then transferred into the microscope for observation.

#### 2.3.3. Pore Structure

The pore structure and the porosity of each sample were determined by mercury injection. The MIP tests were performed using the MicroActive AutoPore V 9600 from micromeritics (Gwinnett, GA, USA), with the applied pressure ranging from 0.67 kPa (0.10 psia) up to 408 MPa (61,000 psia).

### 2.4. Nanostructural Characterization

#### 2.4.1. Establishment of Model

Despite being an amorphous gel substance, C-S-H gel exhibits short-range order at 3.5 nm, as determined by X-ray scattering experiments by Skinner et al. [26]. Numerous researchers have analyzed the structure of this short-range ordered C-S-H gel and found several mineral analogues similar to it. Based on extensive literature comparisons [27,28,29,30], Tobermorite 11 Å was selected as the initial crystal cell model for C-S-H. This study used a calcium-to-silicon ratio of 0.83 and a water-to-silicon ratio of 0.5 [31], with the chemical formula Ca_5_Si_6_O_16_(OH)_2_·2H_2_O being used for molecular dynamics simulation. The simulation software used was Materials Studio 2023, with the selected force field being pcff_interface [32], which has good support for Tobermorite crystals [33]. Zhou analyzed 17 structures of Tobermorite 11 Å with a calcium-to-silicon ratio of 0.83 and found that the further apart the Si-OH groups on the chains, the lower the energy, indicating that the position of hydrogen in the molecular chain significantly affects the overall stability of the structure. Fe [34], building on this research, used geometric optimization and analysis of unit cell parameter variation rates to determine the optimal Si-OH positions. In this study, after reviewing the literature, we chose two configurations with the lowest variation rates for verification and selected the optimal C-S-H structural form based on their performance.

**Figure 3:**
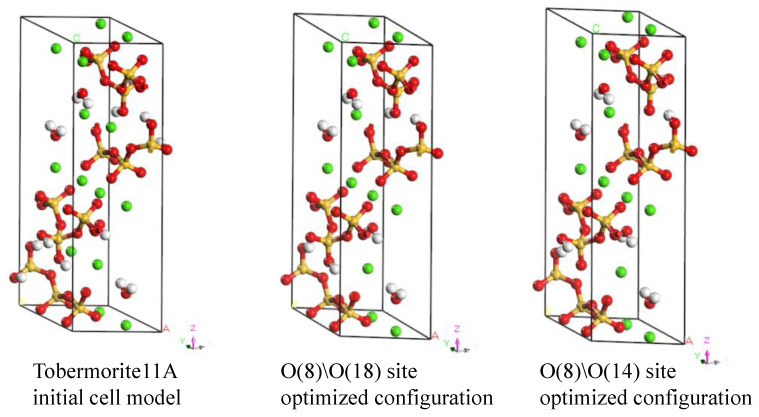
Molecular dynamics simulation structural model (red—oxygen O; green—calcium Ca; orange—silica Si; white—hydrogen H).

**Figure 4:**
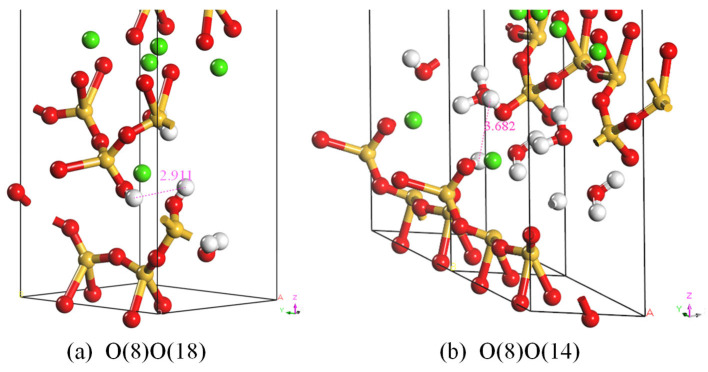
Local molecular structures after geometric optimization.

As shown in Figure 3, this study optimized two configurations based on the initial Tobermorite11 Å structure: the O(8)\O(18) site-optimized configuration and the O(8)\O(14) site-optimized configuration. Subsequently, the Forcite module was employed for geometry optimization with the following settings: quality set to ultra-fine, maximum number of iterations set to 1000, and energy summation method set to Ewald. The optimized molecular cell parameters and total energies are listed in Table 3. The C-S-H, O(8)O(14) configuration exhibits lower energy. Moreover, as illustrated in Figure 4, the distance between hydrogen atoms on adjacent silicate-oxygen chains in configuration (b) is greater, measuring 3.682 Å. These findings indicate that the C-S-H, O(8)O(14) system possesses higher symmetry, reduced steric hindrance, and enhanced structural stability. Therefore, this configuration was chosen as the initial C-S-H cell model for this study.

Upon selecting the appropriate structure, the symmetry module was utilized to orthorhombize and periodize the unit cell. Orthorhombization ensures that the atomic coordinates within the model are independent, thereby simplifying numerous calculations. Periodicity, on the other hand, considers that the interactions between atoms within the model are periodically repeated throughout the crystal. This approach not only reduces the influence of boundary effects on simulation results, but also aligns with the inherent continuity of molecular chains in the crystal structure model. However, to ensure that the model exists independently within the lattice, the crystal must be cut. Post-cutting, geometric optimization was performed using the Forcite module with the following settings: quality set to medium and maximum number of iterations set to 50,000. The optimized model was then subjected to five cycles of annealing within the temperature range of 300–500 K to generate the structure with the lowest potential energy. Subsequently, under standard atmospheric pressure (1 atm), the target system model underwent 5 ns of dynamic relaxation simulation and density optimization using the constant-pressure, constant-temperature (NPT) ensemble. This approach ensures that the model’s density is optimized under realistic conditions, enhancing the accuracy and reliability of the simulation results.

#### 2.4.2. Molecular Dynamics Simulation

Since the software does not inherently support electromagnetic environments, this study utilized a Perl script to establish an alternating electric field to simulate a microwave field environment. The influence of magnetic fields on molecular simulations is negligible, as confirmed by Motohiko et al. [35]. Therefore, the microwave field script content was constructed using Equation (1) [35,36].

(1) $${\tilde{E}}_{x}\left(t\right)=E_{0}\sin\omega t,\;\;\;\tilde{B}=0\;$$

where *E*_0_ signifies the amplitude of the electric field and *ω* denotes the frequency.

The NPT ensemble in molecular dynamics simulations can mimic an isothermal and isobaric environment. Due to the numerous limitations of macroscopic experiments aiming to study microwaves’ non-thermal effects, it is feasible to use computer simulations to create an ideal environment. The dynamic optimization temperatures with and without a microwave field were set to the same value (293 K) to observe the differences in their outcomes, thereby simulating the non-thermal effects of microwaves on C-S-H. Additionally, two higher temperatures (313K and 333 K) were set to simulate the thermal effects of microwaves. It is important to note that the model, after orthogonalization and periodicization, exhibits a layered structure. To ensure that the model would fully absorb the microwave effects, the simulation environment was set to apply the microwave field perpendicular to the interlayer direction.

## 3. Results and Discussion

### 3.1. Pore Structure

During the hydration process of cement, the internal pore structure continuously evolves. Different curing conditions result in varying hydration rates, which lead to different developments in pore structures. The sizes of pores in concrete vary widely and have different impacts on its mechanical properties, dimensional stability, and durability [37]. Pores smaller than 20 nanometers are considered safe; those between 20 and 100 nanometers have minor detrimental effects; pores in the range of 100 to 200 nanometers are deemed harmful; and pores larger than 200 nanometers are highly detrimental [38].

**Figure 5:**
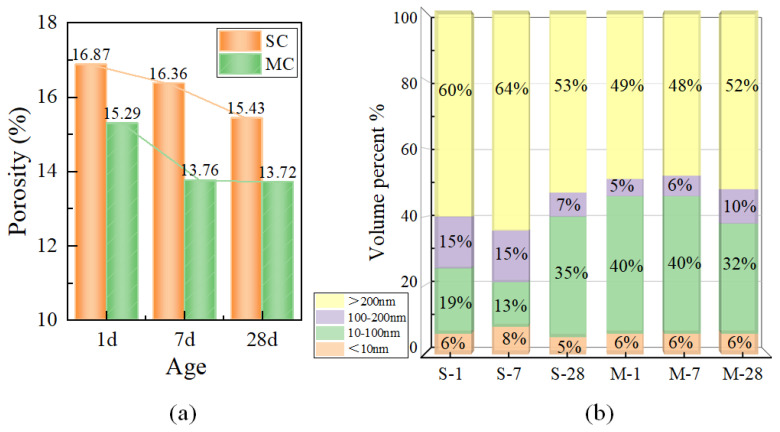
Pore volume and pore size distribution of SC (S-x) and MC (M-x) specimens: (**a**) pore volume, (**b**) pore size distribution.

As shown in Figure 5a, the pore volume of the microwave-cured (MC) specimens is significantly lower than that of the standard-cured (SC) specimens, indicating that microwave curing improves pore structure. According to the trend line of pore volume changes, the pore volume of SC decreased uniformly, with a daily average reduction of 0.085% in the first seven days and 0.044% from day 7 to day 28. In contrast, the pore volume of MC decreased rapidly in the early stages, with a daily average reduction of 0.15%, and underwent a smaller change in the later stages. This suggests that microwave curing affects the rate of pore volume reduction, accelerating the early reduction of pore volume. Additionally, microwave curing accelerates the hydration rate of concrete, leading to minimal total pore volume reduction in the later stages.

Furthermore, as shown in Figure 5b, in the early stages, the pores in SC were primarily distributed in the range of >200 nm, while the pores in MC were mainly distributed in the range of 10–100 nm. This indicates that microwave curing reduces the number of highly detrimental pores and increases the number of less harmful pores. Additionally, the pore size distribution of SC at 28 days shows that as the curing time increases, the pore size within the specimens gradually decreases, reducing the harmfulness of the pores. The amount and the distribution of hydration products gradually converge under the two curing methods, resulting in no significant difference in pore size distribution. This further illustrates that microwave curing does not have a negative impact on the pore structure of concrete materials. Although the proportion of pores larger than 100 nm in SC at 28 days was less than that of MC, the total pore volume of MC was lower, so the total number of pores in this range was still lower in MC compared to SC.

**Figure 6:**
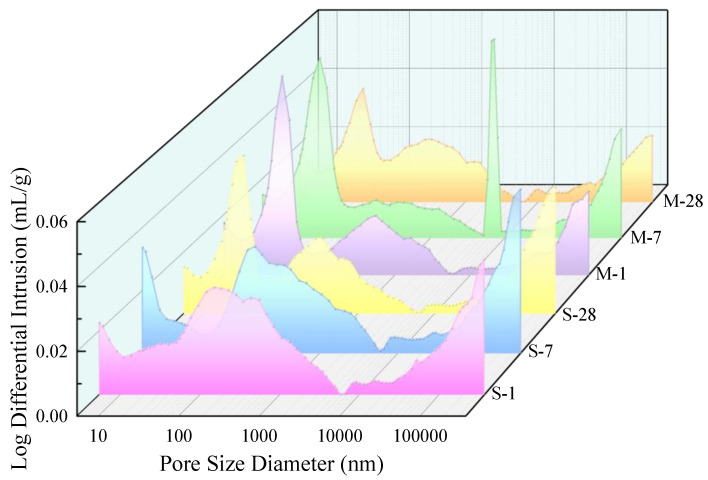
Log differential intrusion vs. pore size of SC (S-x) and MC (M-x) specimens.

As illustrated in Figure 6, it is evident that specimens cured with microwaves exhibit a greater proportion of small-sized pores that are more concentrated, whereas SC specimens display a wider range of pore sizes and larger average pore diameters. The trend lines over time demonstrate that pores in the size range of 100–1000 nm progressively contract to smaller pore sizes within the range of 10–100 nm. The trend of SC-28d is similar to that of MC at all ages, indicating that MC specimens significantly reduce the number of harmful larger pores early on and exhibit superior pore structure characteristics [4,39]. This observation confirms that microwave curing promotes the formation of hydration products, which fill in the pores, resulting in a denser structure [40]. Due to the fact that no air-entraining agents were used in this study, it is possible that air could not be effectively expelled during sample preparation and curing, leading to local pore structure abnormalities. Consequently, a slender and anomalous peak emerged in the curve of the M-7 sample.

### 3.2. XRD Analysis

**Figure 7:**
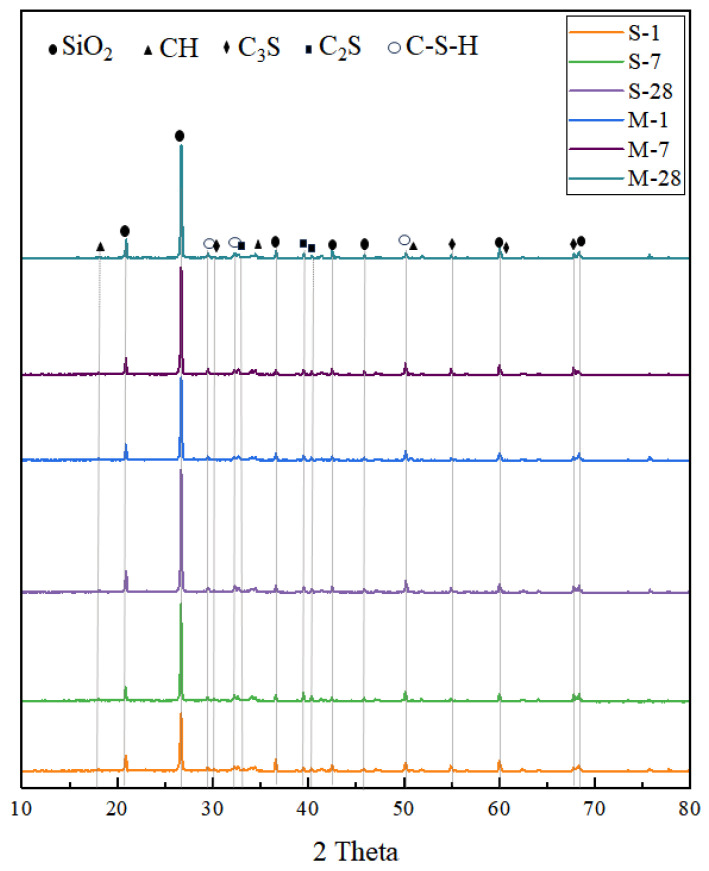
XRD spectra of specimens cured under standard and microwave conditions at different ages (1, 7, and 28 days).

The crystalline phase characteristics of the samples were determined through XRD analysis. Figure 7 presents the XRD spectra of specimens cured under standard and microwave conditions at 1, 7, and 28 days of age. Comparing the results with those of standard card, the phases present in the samples include unhydrated tricalcium silicate (C_3_S), dicalcium silicate (C_2_S), quartz (SiO_2_), hydrated calcium hydroxide (CH), and C-S-H.

Variations in the XRD patterns indicate differing degrees of hydration. Notably, after 7 days, the specimens cured under microwave conditions exhibited higher C-S-H content compared to those cured under standard conditions, suggesting faster hydration under microwave curing. After 28 days, the MC specimens showed lower contents of C3S, C2S, and CH compared to the SC specimens, indicating that microwave curing promotes further hydration and pozzolanic reactions. The reduction in CH content is attributed to the consumption of CH by the pozzolanic reaction involving silica fume [39].

### 3.3. Microstructure

**Figure 8:**
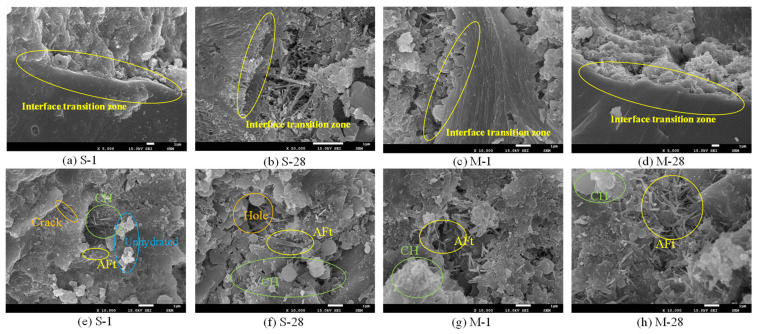
Microstructural morphology of specimens cured under standard and microwave conditions at different ages.

To gain deeper insights into the impact mechanism of microwave curing on the performance of UHPC, this study conducted microstructural characterization of specimens cured under standard and microwave conditions. As shown in Figure 8a–d, microwave curing significantly enhanced the ITZ of UHPC. The ITZ is a weak zone between aggregates and the cement matrix, and its properties directly affect the overall strength and durability of the concrete. Microwave curing, through the action of high-frequency electromagnetic fields, accelerated the hydration reactions between the cement matrix and aggregates, promoted the formation of C-S-H gel, and reduced the porosity and microcracks in the ITZ. Additionally, efficient absorption of microwave energy led to a significant increase in the dielectric constant of the ITZ, indicating an enhanced polarization capacity and further optimization of its densification and mechanical properties. This optimization not only improved the compressive and flexural strengths of UHPC, but also significantly enhanced its impermeability and durability. As depicted in Figure 8e–h, a comparative observation of the microstructures of specimens subjected to different curing conditions revealed that the microstructure of SC specimens (1d) was relatively loose, with evident microcracks and voids. These voids contained a higher proportion of free water and bound water, resulting in a higher dielectric constant [24]. In contrast, the microstructure of MC specimens exhibited greater denseness. Microwave curing accelerated the pozzolanic reactions, consuming a significant amount of CH and leading to a notable decrease in CH content after 28 days. Furthermore, microwave curing promoted the formation of needle-like AFt over 1 day, and these AFt gradually filled the voids over 28 days, forming a dense, interwoven needle-like structure that completely sealed the voids, constructing a tightly cross-linked structure. This demonstrates that microwave curing can optimize the internal void structure and enhance the denseness of the microstructure.

### 3.4. Nanostructure in Molecular Dynamics

#### 3.4.1. Nanoscale Pores in the Structure

Nanoscale pores can be represented by free volume in molecular simulations. Free volume refers to the space within the unit cell that is not occupied by the elements of the model. In isothermal and isobaric environments, when a microwave field is applied to the cement hydration product model, the overall model exhibits strong oscillations along the direction of the electric field. These oscillations compress the gaps between elements, leading to a more homogeneous and dense arrangement of components within the unit cell. Additionally, as the model densifies under microwave action, the squeezed space is expelled from the unit cell due to external pressure, causing the cell parameters to decrease. This indicates that the microwave field promotes the densification of cement hydration products through a physical mechanism (See Table 4). Meanwhile, analysis of Table 4 reveals that as the temperature increases, the compression of space within the unit cell parameters decreases. This phenomenon suggests that thermal effects are not the primary cause of densification in cement hydration products that undergo microwave curing. Instead, the mechanical oscillation effect of the microwave field is likely the main driving force behind densification.

**Figure 9:**
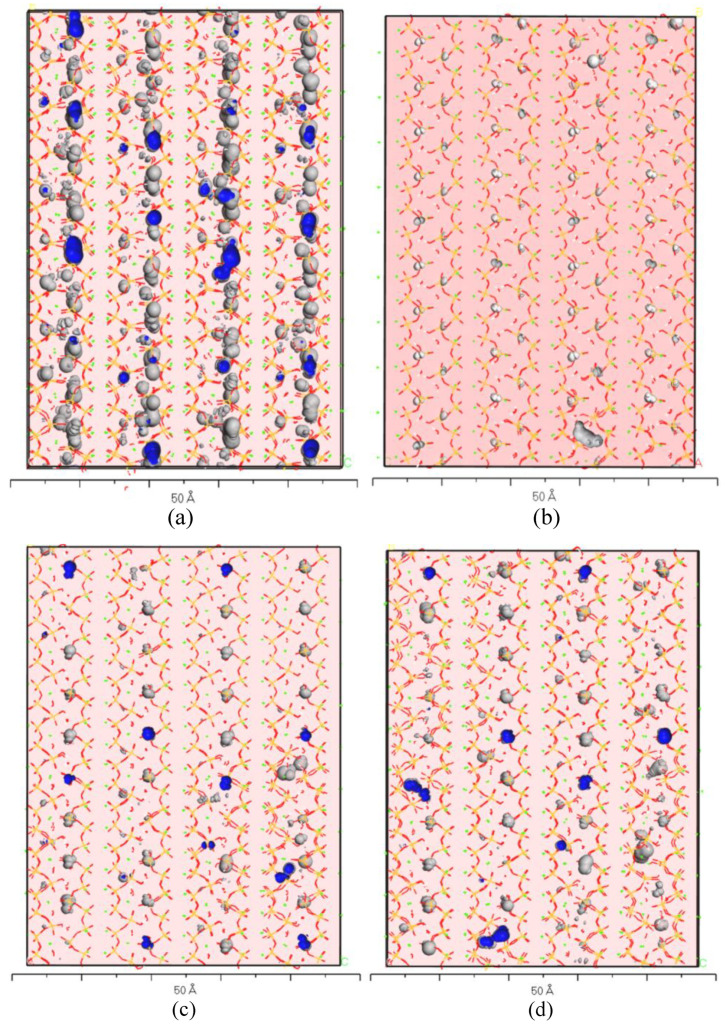
Free volume of C-S-H in different environments and at different temperatures: (**a**) non-MW-293K; (**b**) MW-293K; (**c**) MW-313K; (**d**) MW-333K.

Figure 9 depicts the optimized free volume characterization structures for various models, where gray bubbles represent the pores and blue regions indicate the interior of the bubbles. The visualization reveals that the introduction of the microwave field significantly reduces the pore volume in the models, indicating that microwave treatment helps minimize nanoscale pores in cement hydration products. However, as the temperature increases, the volume of the bubbles in the models increases, and their proportion rises. This suggests that thermal effects not only inhibit the shrinkage of C-S-H unit cells, but also retain more pores within the layered structures [41]. This finding has significant implications for optimizing temperature control in cement hydration processes.

#### 3.4.2. Radial Distribution

To better observe the effects of microwave radiation on the nanoscale structure of C-S-H, simulations were conducted under varying environments and temperatures, including standard curing and microwave curing conditions. Additionally, by comparing the two environments at a temperature of 293 K, the non-thermal effects of microwaves on the structure can be investigated. The RDF, a crucial tool for structural characterization, was utilized to quantify the distribution of interatomic distances and local density variations.

**Figure 10:**
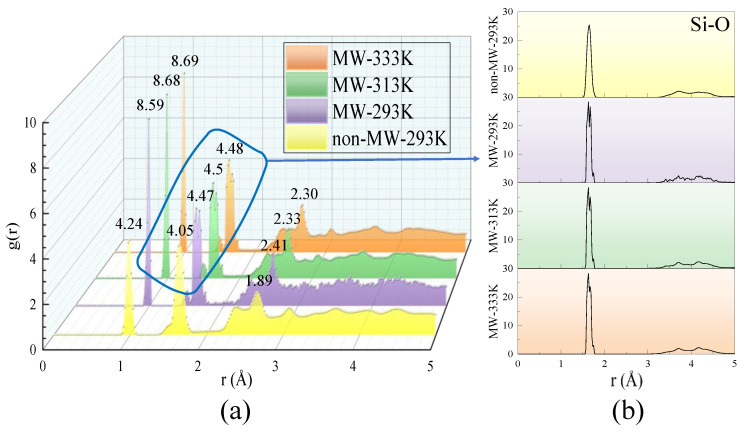
RDF curves at different temperatures with and without microwave fields: (**a**) total; (**b**) Si-O.

As shown in Figure 10a, the introduction of a microwave field results in a significant increase in the peaks of the RDF curves, which are affected to varying degrees. The differences between RDFs with and without microwave fields are more pronounced at the same temperature, while the variations caused by temperature elevation are relatively minor. This observation indicates that the non-thermal effects of microwaves have a more substantial impact on the structure compared to their thermal effects. Notably, at the first peak, located around 1 Å, which corresponds to O-H bonds within the model, the peak height is notably higher than the others, suggesting the presence of a substantial number of water molecules and hydrogen bonds in the system. The microwave field induces a more ordered arrangement of these hydrogen bonds, leading to a sustained increase in peak height. Moreover, water molecules within the model exhibit intense vibrations under the microwave field, indicating that microwaves directly influence hydrogen bond structures by altering the orientation of water molecules.

The non-thermal effects of microwaves primarily arise from the electromagnetic field’s influence on molecular dipole moments. The reorientation of dipole moments leads to changes in molecular alignment, resulting in the splitting or merging of RDF peaks. This phenomenon is particularly evident in Figure 10b, where the RDF peaks under a microwave field exhibit more complex variations, reflecting the multi-layered regulation of the C-S-H structure by microwaves.

### 3.5. Uniaxial Tension and Tensile Fracture Simulation

**Figure 11:**
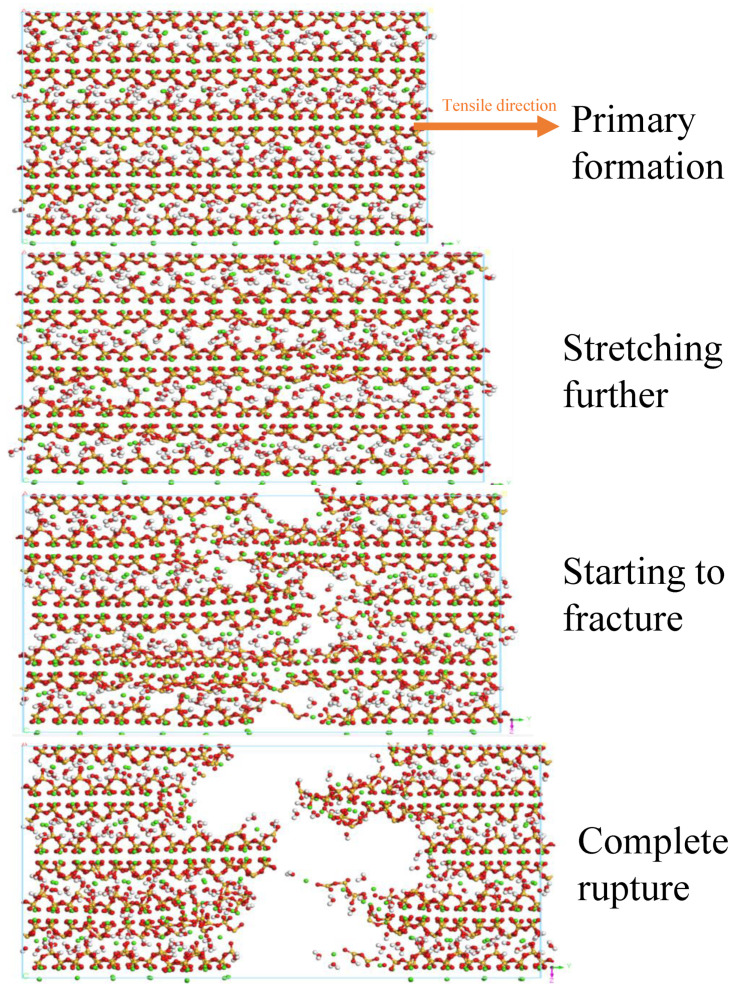
Tensile fracture process of C-S-H model along interlayer direction, parallel to interlayer direction.

To investigate the effect of microwaves on the stress–strain relationship of C-S-H, a uniaxial tension load was applied to the dynamics-optimized model in a direction parallel to the interlayer, with a loading rate of 0.02%. The model was loaded for 30 cycles, continuously elongating until fracture (as shown in Figure 11). During the elastic stage, the uniaxial tension along the interlayer direction mainly resulted in the elongation of Ca-O bonds and fine adjustments in the Ca-O-Si bond angles. This change reflects the local stress distribution characteristics of the C-S-H structure within the elastic domain. As the increasing stress caused progression to the yield stage, the bonding between calcium clusters and silicate chains weakened, leading to the fracture of local Ca-O bonds, thereby initiating microcracks within the C-S-H layers. These microcracks are a direct manifestation of local failure in the C-S-H structure under tensile load. Compared to Ca-O bonds, Si-O bonds have a higher amount of bond energy and require more energy to break, resulting in the long silicate chain network exhibiting a greater load-bearing capacity. Continued loading caused the model’s cracks to progressively expand and propagate, ultimately leading to complete fracture. After fracture, the two parts returned to their original lengths from the elongated state. It is worth emphasizing that the initiation of fracture is indeed associated with the preferential fracture of Ca-O bonds, but the silicate chains are crucial for the overall structural stability.

**Figure 12:**
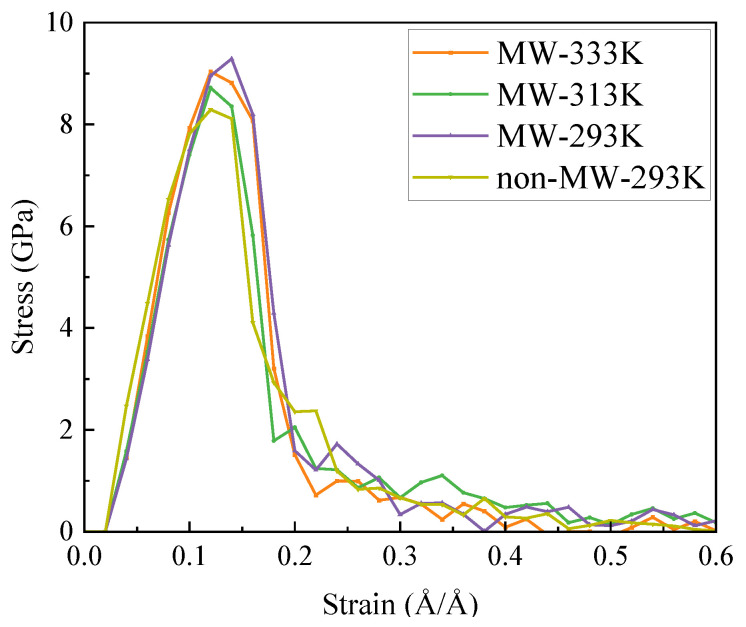
Stress–strain behavior of C-S-H in different environments and at different temperatures.

The tensile strength of the C-S-H model ranges between 8 and 9.5 GPa (as shown in Figure 12). The stress–strain curve does not exhibit a distinct yield stage. The elastic stage is longer in the microwave-exposed model compared to the non-exposed model, delaying the transition to the subsequent stage. The graph clearly illustrates that the strength of the microwave-exposed model is higher, with an increase of up to 1 GPa. This indicates that microwaves enhance the tensile strength to some extent by optimizing the internal structure, demonstrating what is termed “nano-ductility” [11].

## 4. Conclusions

This paper presents a multi-scale study on the microstructural changes of UHPC under microwave curing conditions, ranging from experimental investigations to molecular dynamics simulations. Based on the obtained results, the following main conclusions are drawn:

1. Microwave curing promotes the development of porosity in UHPC during the early stages and optimizes the pore size distribution. The average daily porosity reduction was 0.085% for the SC specimens in the first 7 days, while it was 0.15% for the MC specimens. Compared to the SC sample, the MC sample exhibits a higher proportion of small pores, indicating that microwave curing optimizes the pore structure. Microwave curing accelerates the hydration rate of concrete, resulting in a relatively smaller reduction in total porosity during the later stages. The pore size distribution at 28d demonstrates that microwave curing has no adverse effects on the development of the microstructure of concrete in the later stages.
2. Microwave curing accelerates the formation of hydration products, such as AFt, which gradually fill the voids within the structure and form a dense cross-linked structure within 28 days. The formation of this structure further optimizes the internal void structure of UHPC, enhancing the density of its microstructure and thereby increasing structural strength. The significant reduction in CH content over 28 days suggests that microwave curing also accelerates the pozzolanic reaction process, consuming a substantial amount of CH.
3. By simulating an ideal microwave field environment using computer modeling, it was found that the non-thermal effects of microwaves have a more significant impact on the structure than the thermal effects, providing a theoretical basis for optimizing the curing regime. Additionally, the mechanical oscillation effect improves the orderliness of molecular arrangement, compresses the free space within crystal cells, and promotes the densification of cement hydration products. However, the thermal effects have an adverse impact on pore structure optimization.
4. The microwave field facilitates the enhancement of structural toughness by optimizing the internal architecture of the model, promoting closer interlayer structural connections which, in turn, lead to an increase in tensile strength of up to 1 GPa. Regarding the stress–strain behavior of the field model, the elastic stage is significantly longer, implying that the field model can maintain higher stress levels at the same strain level without entering the next stage.

This study advances understanding of microwave curing mechanisms and provides critical technical insights for the construction industry. By optimizing pore structure, accelerating hydration reactions, amplifying non-thermal effects, and enhancing mechanical properties, microwave curing technology offers significant advantages in energy efficiency, sustainability, and scalability. Future refinement of process parameters tailored to practical engineering needs is anticipated to drive widespread UHPC adoption in green construction, thereby accelerating the industry’s shift toward low-carbon development.

## Figures and Tables

**Table 1:** Physical indicators of quartz sand.

Physical Indices	
Mesh size	40–140
Fineness (μm)	109–380
SiO_2_ (%)	≥97
Fe_2_O_3_ (%)	≤0.1
Apparent density (g/cm^3^)	3.98
Packing density (g/cm3)	1.374

**Table 2:** Proportioning of UHPC mixes.

Constituent	Mass (g)
Cement	800
Quartz sand	880
Silica fume	201
Water	184
PCE superplasticizer	3

**Table 3:** Cell parameters and total energies of molecular configurations optimized with different Si-OH sites.

Cell Parameter	Tobermorite11 Å	C-S-H, O(8)O(18)	C-S-H, O(8)O(14)
a/Å	6.69	5.96	5.20
b/Å	7.39	6.91	6.80
c/Å	22.77	24.63	28.43
α/°	90.00	90.00	90.00
β/°	90.00	90.00	90.00
γ/°	123.46	125.278	131.31
Total energy(kcal/mol)	—	−10,071.2	−10,192.5

**Table 4:** Optimized unit cell parameters of C-S-H under different conditions and at different temperatures.

Lengths (Å)	a	b	c
non-MW-293K	13.44	61.50	45.25
MW-293K	13.36	61.13	44.97
MW-313K	13.36	61.14	44.98
MW-333K	13.39	61.20	45.03

## Data Availability

The original contributions presented in this study are included in the article. Further inquiries can be directed towards the corresponding author.
